# Level of agreement of point-of-care and laboratory HbA1c measurements in the preoperative outpatient clinic in non-diabetic patients who are overweight or obese

**DOI:** 10.1007/s10877-019-00255-6

**Published:** 2019-01-18

**Authors:** Floris van Raalten, Yasmine L. Hiemstra, Noor Keulen, Yoni van Duivenvoorde, Katrin Stoecklein, Evert A. Verhagen, Christa Boer

**Affiliations:** 1 Department of Anesthesiology, Amsterdam UMC, VU University, Amsterdam Cardiovascular Sciences, De Boelelaan 1117, 1081 Amsterdam, The Netherlands; 2 Department of Public & Occupational Health, Amsterdam UMC, VU University, Amsterdam Public Health, De Boelelaan 1117, 1081 Amsterdam, The Netherlands

**Keywords:** Anesthesia, Preoperative screening, Obesity, Glucose, Diabetes

## Abstract

Implementation of point-of-care HbA1c devices in the preoperative outpatient clinic might facilitate the early diagnosis of glycemic disturbances in overweight or obese patients undergoing surgery, but validation studies in this setting do not exist. We determined the level of agreement between a point-of-care and laboratory HbA1c test in non-diabetic patients visiting the outpatient clinic for preoperative risk profiling. Point-of-care HbA1c levels were measured in whole blood obtained by a finger prick (Siemens DCA Vantage HbA1c analyzer) and in hemolysed EDTA blood in the central laboratory (LAB). Bland Altman and Clarke’s error grid analysis were used to analyze the agreement between the point-of-care and laboratory measurements. Patients (n = 49) were 55 ± 11 years old, 47% were male with a body mass index (BMI) of 30.6 ± 3.4 kg/m^2^. The mean HbA1c was 38.1 ± 3.7 mmol/mol or 5.6 ± 0.3%. One patient was diagnosed with a HbA1c indicative for diabetes mellitus (6.7%). Bland Altman analysis revealed a bias of − 0.53 ± 1.81 mmol/mol with limits of agreement of − 4.09 to 3.03 mmol/mol and a bias of − 0.05 ± 0.17% with limits of agreement − 0.39 to 0.28%. The percentage error was 9.2% and 5.9% for HbA1c expressed in mmol/mol and %, respectively. Clarke’s error grid analysis showed that 48 out of 49 measurements were located in area A (98%). Point-of-care HbA1c measurements showed a high level of agreement with the laboratory test in the outpatient setting, and may be used for preoperative risk profiling in patients prone to cardiometabolic complications.

**Trial registration**: Netherlands Trial Register NTR3057.

## Introduction

As a result of the worldwide obesity epidemic, anesthetists are increasingly faced with overweight or obese patients undergoing anesthesia and surgery [1]. A recent study showed that among patients visiting a preoperative screening outpatient clinic, 47.5% had a body mass index exceeding 25 kg/m^2^ [2].

While obesity is a risk factor for the development of postoperative hyperglycemia [3], non-diabetic patients who are overweight are not routinely screened for metabolic abnormalities during their visit to the preoperative outpatient clinic. In particular, routine glucose measurements require a fasting state of the patient, which prohibit broad implementation in the preoperative outpatient setting. Alternatively, circulating glycohemoglobin (HbA1c) levels may be used as an indicator of average blood glucose concentrations over the preceding 2–3 months.

An observational study in presumed non-diabetic patients undergoing gynecological cancer surgery, however, showed that 17.3% of these patients suffered from impaired glucose tolerance or diabetes [4]. In a cohort of 7565 surgical patients 54 years or older, HbA1c measurements revealed that 30% and 37% had HbA1c levels indicative for diabetes or prediabetes, respectively [5]. Moreover, a preoperative elevated HbA1c is associated with higher mean postoperative glucose levels in patients with no diabetic history [6]. A systematic review showed that a high preoperative HbA1c (> 6%) in non-diabetic subjects was associated with an increased risk of overall postoperative complications, but this association may vary among specific populations [7].

Routinely, HbA1c measurements require a laboratory-based blood analysis by a venipuncture. Alternatively, point-of-care HbA1c measurements in blood obtained by a finger prick in the preoperative outpatient setting may facilitate a fast diagnostic and therapeutic work-up in case of a high HbA1c in non-diabetic patients [8]. Moreover, these patients may be referred to an intensive behavioral lifestyle intervention program to improve their preoperative health condition [9]. The Siemens DCA Vantage™ point-of-care HbA1c analyzer is well studied in the diabetic population and validated against laboratory measurements with good results [10, 11], albeit reports of poor level of agreement also exist [12]. The validity of point-of-care HbA1c measurements in patients with an unknown metabolic condition visiting the preoperative outpatient clinic is however unknown. We therefore evaluated the level of agreement between the point-of-care HbA1c against a laboratory HbA1c measurement in non-diabetic patients undergoing elective surgery in the preoperative outpatient setting in order to investigate whether the point-of-care test meets the clinical standards for accuracy.

## Methods

### Study population

This prospective observational study was performed in the preoperative outpatient clinic of the Department of Anesthesiology of Amsterdam UMC, location VU University Medical Centre (Amsterdam, the Netherlands). The study was a substudy of the larger POSitive trial (NTR3057). The POSitive trial was approved by the Ethical Committee of VU University Medical Center, Amsterdam, the Netherlands (Ethical Committee No. NL42863.029.12) on 4 March 2013 and all patients provided written informed consent. The POSitive trial included non-diabetic patients visiting the preoperative outpatient clinic with a body mass index > 25 kg/m^2^ and a HbAc1 > 5.5%.

Data were retrieved between November 2013 and February 2014. During this 4-month period, consecutive patients who were overweight or obese were asked to participate in the study.

Patients were eligible for inclusion when they were 18 years or older, had a body mass index that exceeded 25 kg/m^2^ and were planned for elective surgery. Patients with a known history of diabetes mellitus type I or II were excluded.

### HbA1c measurements

HbA1c measurements took place during the consultation with the anesthetist. The point-of-care (POC) HbA1c was measured using the Siemens DCA Vantage™ analyzer (Siemens Medical Solutions Diagnostics, Tarrytown, NY), which is based on latex agglutination inhibition immunoassay methodology and provides results within 6 min. The system consists of a spectrophotometer and precalibrated, unitized reagent cartridges containing both wet and dry reagents. Specific barcoded cards identify batches of reagents and controls. The immunological reaction uses a monoclonal antibody, and light scattering is quantitated from the absorbance measured at 530 nm, simultaneously with total hemoglobin evaluation using potassium ferricyanide. To measure HbA1c, 1 µL of capillary or venous blood is required that was obtained by performing a finger prick.

After the point-of-care measurements, patients were referred to the laboratory for a venipuncture on that same day in order to determine the HbA1c by a standard laboratory test (LAB) using blood samples collected in EDTA-containing tubes. For the laboratory HbA1c test, blood is hemolysed and injected in a High-performance liquid chromatography (HPLC) column, which separates the stable HbA1c-fraction of other hemoglobin fractions. The HbA1c result is then calculated as a ratio to total hemoglobin by using a chromatogram. HbA1c results measured by the point-of-care test were categorized as normal (HbA1c ≤ 5.5%), prediabetes (HbA1c 5.6–6.4%) or diabetes (HbA1c ≥ 6.5%).

### Other study parameters

During the preoperative visit, biomedical measurements were performed; i.e. body height, body weight, waist circumference and blood pressure. Other study parameters included patient demographics and the current medication use.

### Data analysis

The sample size was based on the general recommendations of Altman of at least 50 subjects in a methods comparison study [13]. Data were analyzed using SPSS 22.0 (IBM, New York, USA) and expressed as mean ± standard deviation (SD), median with interquartile range or frequencies. All data were tested for normality. Independent sample T-tests were used to see if there were any differences in baseline characteristics between men and women.

The level of agreement between the POC and laboratory HbA1c measurement was evaluated using a Bland–Altman analysis (GraphPad Prism 6.0, La Jolla, CA, USA). The Bland–Altman analysis provided the bias, SD of the bias, and limits of agreement between both methods. Agreement was calculated from the bias and SD of the bias (1.96 × SD of bias/average of control). According to the National Glycohemoglobin Standardization Program the limits of agreement must fall within 0.75% HbA1c and the coefficient of variation must not be statistically significant above 3% [14].

A Clarke’s error grid analysis was used to demonstrate the agreement and clinical feasibility of the POC test. The scatter plot was divided into three zones, the clinical relevance of the bidirectional (dis)agreement between the two methods. Zone A (green) shows the clinical acceptable area with an acceptance of 10% deviation between the two methods [15]. Zone B (yellow) represents an error > 10% and in this area, hyperglycemia is over- or underestimated. Zone C (red) represents a clinically unsafe zone in which hyperglycemia is severely under- or overestimated with a high risk for inadequate clinical decision-making [16]. More than 95% of the values should be in zone A and none in zone C for the POC-method to be clinical acceptable, reflecting a P-value < 0.05. Ten percent deviation from the golden standard (i.e. laboratory HbA1c measurement) was considered clinically acceptable. An alpha level of 0.05 was defined as a significant test result.

## Results

### Patient characteristics

Point-of-care and laboratory HbA1c measurements were performed in a total of 50 patients. One patient was excluded from final data analysis because laboratory results were absent. Table 1 provides an overview of the patient characteristics for the study population. Patients were on average 55 ± 11 years old with an average body mass index (BMI) of 30.6 ± 3.4 kg/m^2^. The mean laboratory HbA1c values were 38.1 ± 3.7 mmol/mol or 5.6 ± 0.3%.

**Table 1:** Patient characteristics

	Study population (N = 49)
Males/females (n)	28/21
Age (years)	55 ± 11
Height (cm)	173 ± 9
Weight (kg)	91.8 ± 10.6
Body mass index (kg m^−2^)	30.6 ± 3.4
Waist circumference (cm)	103.9 ± 8.7
Males	105 ± 9
Females	102 ± 9
Systolic blood pressure (mmHg)	140 ± 14
Diastolic blood pressure (mmHg)	85 ± 9
HbA1c (mmol/mol)	38.1 ± 3.7
HbA1c (%)	5.6 ± 0.3

Data are expressed as mean ± SD for 49 patients

*HbA1c* haemoglobin A1c protein

### Level of agreement between the POC and laboratory HbA1c

**Fig. 1:**
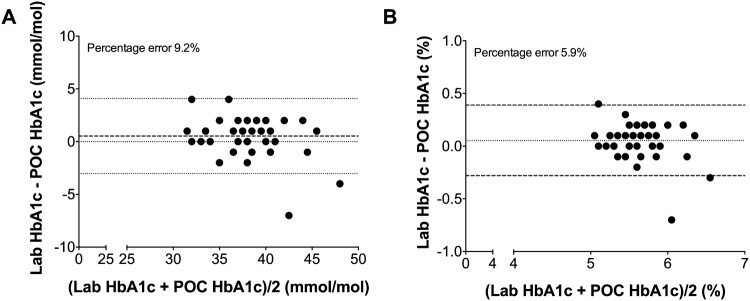
Bland Altman analysis for paired point-of-care (POC) or laboratory (LAB) HbA1c measurements in mmol/mol (**a**) and % (**b**)

Bland–Altman analysis for the level of agreement between the POC analyzer and the standard laboratory test is shown in Fig. 1. The bias for the POC HbA1c in mmol/mol was − 0.53 ± 1.81 and limits of agreement ranged from − 4.09 to 3.03 mmol/mol (panel A). Panel B shows that bias for the HbA1c in % was − 0.05 ± 0.17% with limits of agreement ranging from − 0.39 to 0.28%. The percentage error was 9.2% and 5.9% for HbA1c expressed in mmol/mol and %, respectively.

### Clarke’s error grid analysis

**Fig. 2:**
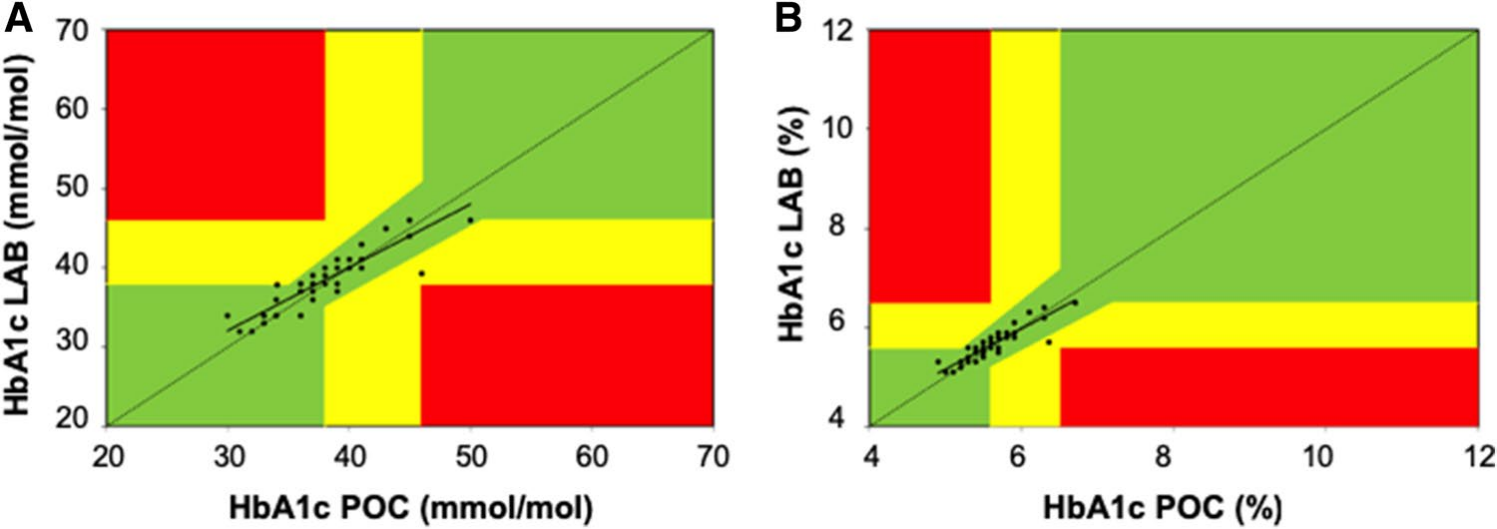
Clarke’s error grid analysis of for the level of agreement between the POC and LAB HbA1c tests expressed as mmol/mol (**a**) or % (**b**). Zone A (green) shows the clinical acceptable area with an acceptance of 10% deviation between the two methods. Zone B (yellow) represents an error > 10% and in this area hyperglycemia is over- or underestimated with possibly changing clinical decision-making. Zone C (red) represents a clinically unsafe zone in which hyperglycemia is severely under- or overestimated with a high risk for inadequate clinical decision-making

**Fig. 3:**
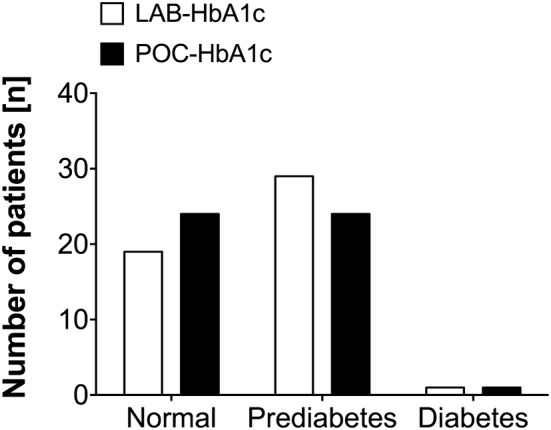
The relative number of patients with normal HbA1c values (≤ 5.5%), prediabetes (5.6–6.4%) and diabetes (≥ 6.5%)according to the laboratory or point-of-care HbA1c test method

Figure 2 shows the error grid analysis for the relative deviation between POC-HbA1c and LAB-HbA1c in mmol/mol (panel A; two patients in zone B) and % (panel B; one patient in zone B). Figure 3 shows the number of patients with normal HbA1c levels, prediabetes or diabetes as found by the laboratory and point-of-care measurement. In one patient, a HbA1c value of 6.5% and 6.7% was found by the laboratory and point-of-care test, respectively, and this patient was referred for further diagnosis of diabetes mellitus to the genal practitioner.

## Discussion

This study demonstrates that the agreement between the point-of-care HbA1c and the central laboratory HbA1c tests in non-diabetic, obese patients or patients who are overweight in the preoperative outpatient setting meets the clinical standards for accuracy. Therefore, we consider the use of the point-of-care HbA1c test valid and feasible to implement in the preoperative evaluation of patients scheduled for elective surgery.

Multiple studies demonstrate the relevance of glycemic monitoring in patients who are overweight or with obesity for the screening of prediabetes or diabetes. In a large study, Sheehy et al. showed that 24% of insured, elective surgery patients with a recent primary care visit had either undiagnosed diabetes mellitus, or impaired fasting glucose (IFG) discovered on the day of surgery [17]. An observational study in non-cardiac surgery patients by Abdelmalak et al. [18] showed that 10% of non-cardiac surgery patients had undiagnosed diabetes mellitus, and 11% impaired fasting glucose levels. From these observations, it could be argued that the preoperative appointment with the anesthetist is an opportune moment for identifying patients with an increased cardiometabolic risk.

The preoperative use of a point-of-care HbA1c measurement as described in the present study has two advantages. First, the point-of-care modality provides anesthetists the opportunity to perform a fast screening of patients who have a risk profile for cardiometabolic alterations. Second, HbA1c measurements have the advantage over an oral glucose tolerance test or plasma glucose testing that they can be performed in non-fasting patients [19]. In case of a high HbA1c during the preoperative screening, a patient can be referred to the hospital laboratory and diabetes department or general practitioner for subsequent diagnostic work-up. In particular, many patients with diabetes are undiagnosed [20], and HbA1c measurements during the preoperative screening may provide an important window for the identification of prediabetes and the institution of interventions that prevent progression to diabetes [21]. Finally, the method is less invasive than tests that require a venipuncture and off-site analysis.

It remains questionable whether high preoperative HbA1c levels in presumed non-diabetic patients are association with unfavorable outcome. In a systematic review, six retrospective and prospective cohort studies were evaluated whether preoperative HbA1c might be indicative for non-diabetic patients at risk of postoperative complications [7]. HbA1c cut-off levels for the prediction of complications ranged from 5.7 to 7%, showing that higher HbA1c levels were related to 30-day postoperative complications, including acute kidney injury, cardiovascular and pulmonary complications, but not with infections [7]. A German study in patients undergoing coronary artery bypass grafting procedures that were analyzed for fasting plasma glucose showed that patients with previously undiagnosed diabetes mellitus were more at risk for postoperative complications than patients with known diabetic disease [22]. These findings argue for broad screening for impairment of glycemic control during the anesthesia screening visit.

The reliability of point-of-care HbA1c measurements in general clinical practice has been debated due to the high variation regarding the accuracy of various point-of-care HbA1c devices. However, the Siemens DCA Vantage™ as used in the present study, was one of the two devices that were found to meet the criteria for accuracy set by the National Glycohaemoglobin Standardization Program (NGSP) [14, 23]. According to the NGSP criteria the limits of agreement must fall within a 0.75% margin and the coefficient of variation must not be statistically significant above 3% in order for POC measurements to be accurate [14]. This is the first study evaluating point-of-care HbA1c measurements in non-diabetic patients visiting the outpatient clinic for preoperative risk profiling. Our results demonstrate that the use of the Siemens DCA Vantage™ analyzer for point-of-care HbA1c measurements meets the criteria set by the NGSP for accuracy and clinical feasibility. Moreover, The Clark’s error grid analysis demonstrates that the use of the Siemens DCA Vantage™ analyzer for point-of-care HbA1c measurements meet the standards for clinical feasibility with < 10% deviation between laboratory and point-of-care measurements.

From our study, we conclude that the Siemens DCA Vantage™ analyzer for HbA1c measurements is valid and feasible to implement in non-diabetic, obese patients who visit the preoperative screening outpatient clinic. In our small patient population with borderline or mild obesity we have shown that the implementation of preoperative HbA1c measurements resulted in the diagnosis of deviating HbA1c values. In our larger POSitive trial, a number of patients were diagnosed with diabetes during resulting work-up and their procedure subsequently postponed.

Our findings suggest that point-of-care HbA1c measurements might be valuable in identifying modifiable risk factors in patients undergoing surgery visiting the preoperative outpatient clinic, and may facilitate the promotion of preoperative prehabilitation and patient optimization programs by anesthetists.
